# *Escherichia coli* resistance mechanism AcrAB-TolC efflux pump interactions with commonly used antibiotics: a molecular dynamics study

**DOI:** 10.1038/s41598-024-52536-z

**Published:** 2024-02-01

**Authors:** Brooke L. Smith, Sandun Fernando, Maria D. King

**Affiliations:** Aerosol Technology Laboratory, Biological and Agricultural Engineering Department, Texas A&M University, College Station, TX 77843 USA

**Keywords:** Virtual drug screening, Bacteria, Proteomics

## Abstract

While antibiotic resistance poses a threat from both Gram-positive bacteria (GPB) and Gram-negative bacteria (GNB), GNB pose a more imminent public health hazard globally. GNB are a threat to growing antibiotic resistance because of the complex makeup of the membrane. The AcrAB-TolC efflux pump is a known resistance mechanism of *Escherichia coli (E. coli)* cells. This study utilized molecular dynamics modeling to visualize some of the changes occurring at a molecular level when airborne bacteria are exposed to stress and antibiotics. This study was conducted to build upon previous experimental research showing that there is an increase in antibiotic resistance and efflux pump activity when exposed to aerosolization. AcrB and AcrAB-TolC proteins were simulated under standard and increased pressure to compare the effect of aerosolization on the binding to the three different antibiotics (puromycin (PUY), ampicillin (AMP) and sulfamethoxazole-trimethoprim (SXT)) to the AcrB binding site. Analysis such as root-mean-square deviation of atomic positions and root-mean-square fluctuation, the opening of TolC, and the significant molecular mechanics with generalized Born and surface area solvation (MM-GBSA) scores associated with specific ligands were recorded. Resistance in experimental data indicated a relationship between the docking scores and some ligand–protein interactions. Results showed that there was more flexibility in the proteins within simulations conducted under standard pressure for the AcrB protein and the full tripartite complex AcrAB-TolC, showing that increased pressure causes more rigidity. MM-GBSA scores, used to calculate the free energy of ligand–protein binding, did not show a significant change, but interestingly, the strongest MM-GBSA scores were for ligands that moved to another binding pocket and did not result in resistance or opening of the efflux pump. However, the ligand moved from the binding site and did not cause the opening of TolC to increase significantly, whereas PUY and AMP were bound to the binding site for the duration of all simulations. AMP ligands under increased pressure showed the largest change in opening of the TolC efflux pump and aligns with experimental data showing *E. coli* cells had the most resistance to AMP after aerosolization. These results, in addition to other real-time changes such as OM proteins and mutations of targets within the cell, could be used to delineate and mitigate antibiotic resistance mechanisms.

## Introduction

Antibiotic resistant bacteria caused approximately 1.27 million deaths and five million associated deaths in 2019 globally. In the United States antibiotic resistant bacteria caused approximately three million infections and 35,000 deaths, according to the Centers for Disease Control and Prevention^1^. Both pathogenic and nonpathogenic bacteria can be classified into two groups: Gram-negative bacteria (GNB) and Gram-positive bacteria (GPB). The World Health Organization (WHO) releases three lists of priority pathogenic bacteria, Priority Level 1 being the most critical. The only bacteria in Priority Level 1 are GNB^2^. While antibiotic resistance poses a threat from GPB and GNB, GNB pose a more imminent threat globally. GNB are a threat because of the makeup of the membrane of the GNB. GNB have an inner membrane surrounding the cell, followed by a thin layer of peptidoglycan and a selectively permeable outer membrane containing an inner phospholipid layer and outer lipopolysaccharides that are negatively charged and interact with the cell’s outer environment. GPB consist of an inner membrane surrounded by a thick layer of permeable peptidoglycan^3^. GNB have an inner membrane (IM), a thin cell wall in comparison to GPB, and an outer membrane (OM)^4,5^.

Mechanisms GNB utilize to evade the effects of antibiotics include acquired, adaptive, and intrinsic resistance. Acquired resistance occurs when bacteria develop resistance from acquisition of resistance genes from extracellular sources or acquire a mutation^6^. Adaptive resistance occurs when gene expression is up or downregulated in the presence of environmental stressors^6,7^. Intrinsic resistance and the expression of antibiotic-inactivating enzymes and non-enzymatic paths can turn to intrinsic resistance after mutations over generations of bacteria. Four mechanisms of intrinsic resistance are limiting drug uptake, modifying drug targets, inactivating drugs, and active drug efflux. These intrinsic changes include the expression of antibiotic-inactivating enzymes, efflux pumps, permeability, or target modifications. The most common uses of intrinsic resistance in GNB are changing permeability of the OM and changing the activity of efflux pumps^4,8^. While efflux pump activity tended to be more intrinsic Smith and King show a case of overlap in adaptive and intrinsic resistance for *E. coli* (a common GNB pathogenic simulant) efflux pumps in which some samples up-regulated the expression of efflux pumps and seemed to express more resistance to antibiotics after being introduced to environmental stress but still possessed the intrinsic ability of resistance to some antibiotics without adapting to its environment^9–11^.

Efflux pumps are a source of multidrug resistance because they can efflux a variety of antibiotics and substrates before they effectively reach and neutralize their targets. The AcrAB-TolC multidrug efflux pump is a resistance nodulation cell division (RND) tripartite protein, having an outer membrane protein (OMP), inner membrane protein (RND), and membrane fusion (MFP) commonly found in GNB *E. coli* and *Pseudomonas aeruginosa*. The AcrAB-TolC efflux pump has a closed apo state in which the Tol-C region is a barrier to the outside of the cell. There is a binding site for ligands in the AcrB region (in the RND). An antibiotic binding to the binding site can cause conformational changes between the AcrB, AcrA and TolC regions of the efflux pump which results in the pump opening in the Tol-C region^12–16^. The AcrB protein determines if the efflux pump will dispose of the substrate and specifically in *E. coli* the pump rotates in conformational changes to alleviate the drug or substrate^17^. The stoichiometric ratio of AcrB, AcrA, and TolC is 3:6:3 respectively. The three identical chains of the AcrB homotrimer contain a transmembrane and a periplasmic domain and the TolC homotrimer contains primarily an α-helical periplasmic domain and a small β-barrel domain. AcrB and TolC regions are connected by six AcrA promoters as a trimer of dimers in the assembled pump^17^. The binding pocket in the AcrB protein was detailed in a study conducted by Wang et al.^12^ which was utilized in this study. Puromycin was used as a control because of the reproducibility of binding puromycin to the specific binding pocket detail in the study conducted by Wang et al.^12^. The three antibiotics used to simulate the activity of the AcrAB-TolC efflux pump were puromycin (PUY), a protein synthesis inhibitor, ampicillin (AMP), a cell wall synthesis inhibitor, and sulfamethoxazole-trimethoprim (SXT), a DNA synthesis inhibitor^18–20^.

Upregulation of *acr*AB, associated with the overactivity of the AcrAB-TolC efflux pump, was dependent on the overexpression of *mar*R or *acr*R genes. Smith and King showed that prior resistance under optimal environmental conditions was not necessary to be expressed after stressors were introduced^21,22^. This concept can be applied to this current research.

Many efflux pump inhibitors (EPI) are under research and development because the AcrAB-TolC efflux pump and other efflux systems are known to cause antibiotic resistance^23^. A key area of the dynamics of the efflux pump and EPI discovery that has not been explored is its relation to bioaerosols. The functioning of the AcrAB-TolC efflux pumps should be considered in relation to the stress response of bacterial cells after aerosolization. Few other studies have shown that a change in pressure can affect other types of cells, such as rat and human cancer cells^24^. A key application is that pathogenic bacteria that are airborne, in hospitals’ heating ventilation and air conditioning (HVAC) systems, become exposed to aerosolization stress and transmit in ways that could infect vulnerable patients. There are many requirements hospitals must follow to have operating HVAC systems; however, little work has been done to monitor the effect of HVAC systems triggering antibiotic resistance in bacteria^25,26^. After experimentation to model the effects of aerosolization due to HVAC, Smith and King^21^ found that *E. coli* MG1655 cells expressed the most antibiotic resistance to ampicillin (AMP) and the least resistance to sulfamethoxazole-trimethoprim (SXT) after aerosolization. After aerosolization *E. coli* cells also showed decreased culturability and an upregulation of specific antibiotic resistance genes including the overactivity of AcrAB-TolC efflux pumps. Molecular dynamics simulations were utilized to visualize the changes of the AcrAB-TolC efflux pump when exposed to the environmental changes of aerosolization. AcrAB-TolC was tested with standard pressure for control samples and increased pressure of 55″ H_2_O after being introduced to aerosolization stress^11,21^. Smith and King^21^ found a 25% increase in resistance in bioaerosols after aerosolization indicating that the stress of aerosolization caused mechanistic changes in the *E. coli* cells to respond to the changing environment.

This study simulated a common antibiotic resistance mechanism AcrAB-TolC efflux pump after experimental data was found by Smith and King^21^ that the increase of stress from aerosolization caused an increased expression of antibiotic resistance mechanisms. Smith and King^11^ also showed that the stress of aerosolization increased efflux pump activity. This study was completed to add to previous research to visualize the changes occurring on a molecular level when environmental stressors impact *E. coli* cells. This study compares the protein activity in a controlled environment with standard conditions and increased pressure the *E. coli* cells experienced during aerosolization. This along with future studies to simulate other changes in the environment will help scientists plan HVAC systems and building designs to impose the least amount of stress on bioaerosols and decrease transmission of antibiotic resistant bacteria in indoor spaces.

## Methods

### Experimentation

A low cutpoint wetted wall cyclone bioaerosol collector (LCP-WWC), with 50% collection efficiency for particles < 300 nm using an aerosol sampling flow rate of 300 L/min at 55″ H_2_O pressure drop and a continuous liquid outflow rate of about 0.2 mL/min, was used to collect the laboratory strain *Escherichia coli* MG1655. *E. coli* cells were aerosolized using a six-jet Collison Nebulizer operating at 20 psi to create a constant stream of aerosolized bacterial cells. The cells were simultaneously collected by the LCP-WWC for 10 min with Tween-20 surfactant as a support liquid and amended with phosphate buffer saline (PBS). Each sample was quantitated during a 15-day archiving period after aerosolization for culturable counts (CFUs) and gene copy numbers (GCNs) using microbial plating and whole-cell quantitative polymerase chain (qPCR) reaction, respectively. The samples were analyzed for protein composition and antimicrobial resistance using protein gel electrophoresis and disc diffusion susceptibility testing. Smith and King^21^ detailed experimentation methods and results. Preliminary results were also obtained from Smith and King^11^, who first showed that aerosolization triggered an antibiotic resistance response.

### Antibiotic resistance development

After collection, the samples were diluted 10 × in Luria Bertani (LB) Broth (BD BBL™, Becton, Dickinson and Co., Franklin Lakes, NJ, USA) and incubated overnight at 37 °C. The next day, 100 µL of each sample was plated on Mueller–Hinton agar for the disc diffusion test. Sensi-Disc dispenser (BD BBL™ Sensi-Disc™, Becton, Dickinson and Co., Franklin Lakes, NJ, USA) was used to place eight antimicrobial susceptibility test paper discs on each plate, impregnated with commonly used antibiotics (30 µg of Tetracycline (TE-30), 10 µg of Ampicillin (AM-10), 30 µg of Cephalothin (CF-30), 10 µg of Gentamicin (GM-10), 10 µg of Imipenem (IPM-10), 5 µg of Ciprofloxacin (CIP-5), 23.75/1.25 µg of Sulfamethoxazole-Trimethoprim (SXT) and 75 µg of Cefoperazone (CFP-75)^11^.

### Molecular docking

**Figure 1:**
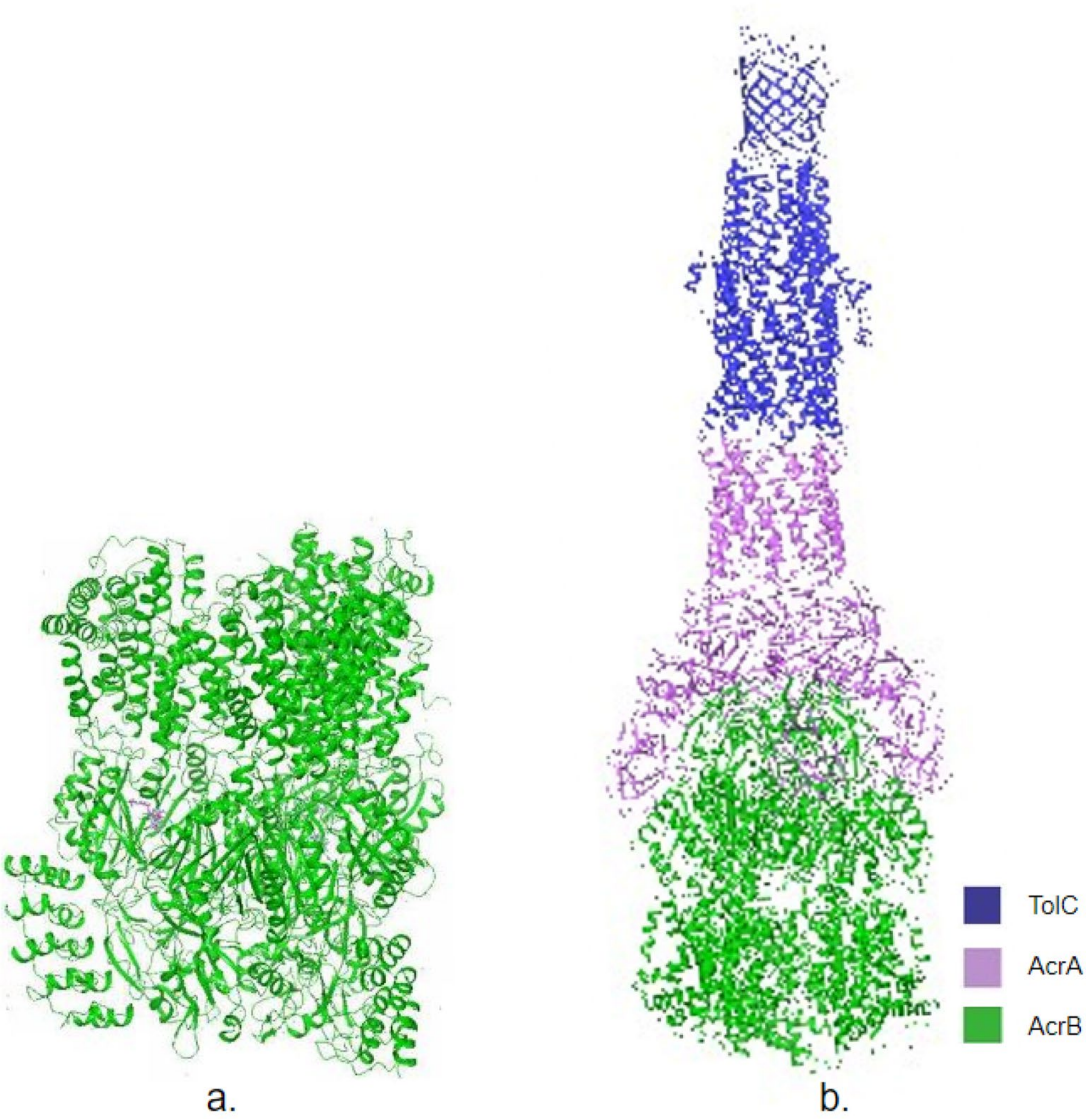
Shows (**a**) the AcrB protein and (**b**) the AcrAB-TolC efflux pump proteins.

The binding of ligands (PUY, AMP, and SXT) to the AcrB binding site of the tripartite complex AcrAB-TolC (shown in Fig. 1) was performed using the Schrödinger Glide platform (Schrödinger Glide)^27^. The AcrB protein (PDB ID: 5nC5) and full efflux pump complex AcrAB-TolC (PDB ID: 5O66) were prepared using the Schrödinger’s Protein Preparation Wizard (Schrödinger Wizard) after downloading the structures from RCSB PDB website^28,29^. The nine ligands used in this study ((Puromycin (PUY), Ampicillin (AM), Cefoperazone (CFP), Cephalothin (CF), Gentamycin (GM), Ciprofloxacin (CIP), Tetracycline (TE), Sulfamethoxazole trimethoprim (SXT), and Imipenem (IPM) were downloaded using Zinc15Docking Sterling and Irwin and prepared using the Schrödinger’s LipPrep tool (Schrödinger Glide)^27,30^. Each of the nine ligands were docked to the AcrB binding site in the protein using the Schrödinger’s Ligand Docking tool (Schrödinger Glide)^27^. The Glide docking score is an empirical value of the binding free energy of the ligand utilizing the electrostatic force field and the van der Waals energy. The Emodel score is calculated using the GlideScore, the internal ligand strain (Einternal), and the Coulomb and van der Waals energy when docking^31^.

### Molecular dynamics simulations

After docking the Schrödinger Desmond platform (Desmond MD)^32^ was used to prepare the setup for the MD simulation. There were four different cases of simulations. The AcrB protein was simulated under standard pressure and increased pressure that the cells experienced after aerosolization^21^. The system charge was neutralized with Na or Cl ions and a 0.1 M concentration of NaCl. The system was built with the Simple Point-Charge (SPC) solvent model. The boundary conditions were set as an orthorhombic box with a buffer distance of 10 Å. The system was built with the Simple Point-Charge (SPC) solvent model. The docked protein and ligand complexes were exported from Glide. The C and N termini of the protein were capped to stabilize the protein structure. All the missing hydrogens were added, and the hydrogen bonds were optimized. The strained minimization was performed with the OPLS3e force field^33^. Each simulation was performed with Desmond’s default relaxation protocol. The standard pressure simulations for AcrB and AcrAB-TolC complexes were completed at 300 K and 1.01325 bar pressure for 100 ns and 10 ns, respectively, once per case. Three ligands per simulation were present to mimic a more natural environment that would contain multiple ligands. Shorter simulations (10 ns) were conducted initially for the full AcrAB-TolC complex because simulating the large size of the protein was computationally intensive. Once the binding site was confirmed, longer (100 ns) simulations were conducted with the AcrB protein. The increased pressure simulations for AcrB and AcrAB-TolC complexes to simulate stress during aerosolization were completed at 300 K and 1.150112 bar pressure for 100 ns and 10 ns, respectively. Post-simulation trajectory analyses were performed by Schrödinger Simulation Interactions Diagram tool to show the stability and flexibility of the protein, including complex root-mean-square deviation of atomic positions (RMSD) and ligand/protein root-mean-square fluctuation (RMSF) complex interactions (Desmond MD)^32^.

### Calculation of binding free energy

The binding interactions of peptide-protein complexes can be calculated using (MM-GBSA) binding energy method^33,34^. The primary MM-GBSA uses the VSGB 2.0 dissolution model with the OPLS3e force field. The docking poses of the AMPs on the receptor were evaluated by calculating the total binding free energy using Schrödinger Prime. Prime MM-GBSA calculations gave complex binding energies, which validated the performance of the current binding conformation. The Schrödinger Prime calculates the energy of the AMP-protein system via MM-GBSA using the Desmond simulation trajectory. From the entire 100 ns simulation, the last 50 ns trajectory was chosen for the energy calculation. The free energy of binding ΔG_bind_ was calculated as the energy of the receptor-ligand complex minus the energy of the receptor alone and the ligand alone, as follows^33^:

$$\Delta {\text{G}}_{{{\text{bind}}}} = {\text{ E}}_{{{\text{complex}}({\text{minimized}})}} - (E_{{ligand\left( {minimized} \right)}} + \, E_{receptor(minimized)} )$$

### Calculation of efflux pump opening

The distance of the AcrB and TolC opening was measured between D-47 and each of the six aspartate residues D-371 and D-374 on each chain (A, B, and C), respectively^35,36^. These changes were calculated and averaged using the Measure tool (Desmond MD). The statistical significance between the changes in the TolC opening was calculated and averaged. The first frame was not considered because the protein was not in an apo (closed) position.

### Statistical analysis

Statistical significance was compared by evaluating several comparisons. First, PUY was compared under standard conditions and increased pressure. Similarly, AMP and SXT were also compared under standard conditions and increased pressure. The means were compared using various *t*-tests to determine statistical significance using Rstudio (Rstudio 2022.12.0 + 353, Posit, PBC, Boston, MA, USA). The first null hypothesis stated that the mean distance of the beta-barrel opening at the beginning of the simulation is equal to the distance at the end of the simulation under increased pressure.

## Results

**Figure 2:**
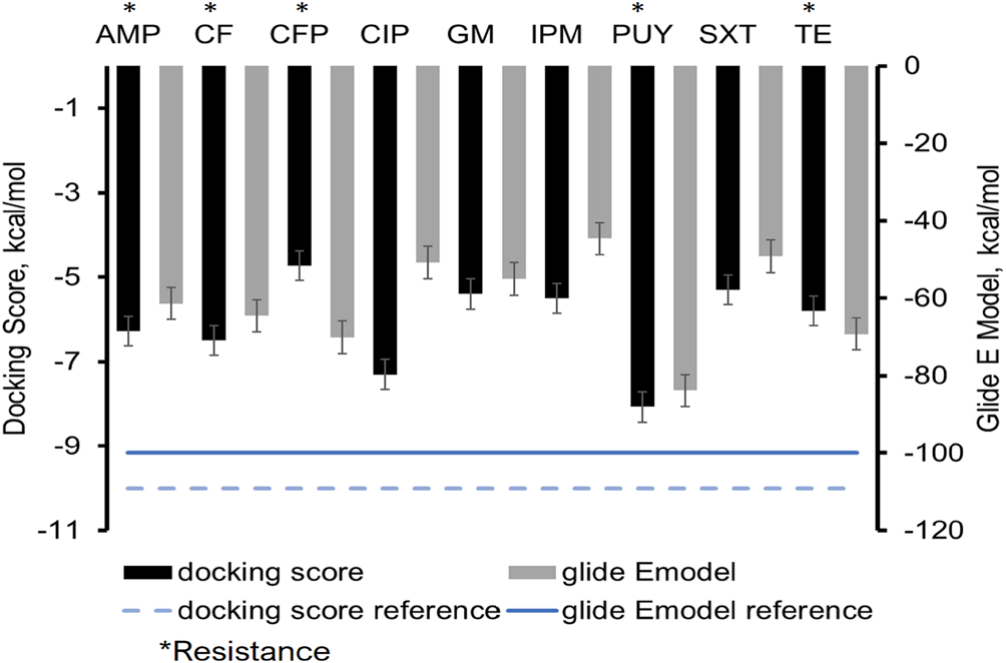
Shows the docking score (black), glide Emodel (gray) score, and reference for desired scores for docking (blue dash line), and glide Emodel (blue line). The antibiotics *E. coli* cells developed the most resistance to were all less than  − 60 kcal/mol Glide E model scores including ampicillin (AMP), cephalothin (CF), cefoperazone (CFP), puromycin (PUY), and tetracycline (TE). The least resistance developed were imipenem and sulfamethoxazole-trimethoprim (SXT).

The docking scores of the nine antibiotics tested and the level of resistance after experimentation are shown in Fig. 2. Although not consistent across all cases, there is a discernible trend between docking and glide Emodel scores and the level of resistance. The most negative docking scores and glide Emodel scores for antibiotics that the *E. coli* expressed the most resistance to after aerosolization. The antibiotics with the most negative Glide Emodel scores include puromycin, cefoperazone, tetracycline, cephalothin, and ampicillin. The least resistant and least negative Glide Emodel scores were ciprofloxacin, gentamycin, sulfamethoxazole-trimethoprim and with the lowest being imipenem. Those showing some resistance had Glide Emodel scores of less than − 60 kCal/mol.

### Ligand interactions

Interestingly, SXT migrated to a different site (approximately 15 Å) from the original binding site and was bound more tightly to this alternative site.

Simulation interactions were performed to identify ligand-residue contacts using the Simulation Interaction Diagram tool in Desmond, Contacts with residues for more than 30% of the simulation time (i.e., 30% interaction fraction) were noted. Puromycin established four polar interactions (at least 50% of the time) with threonine (THR) and asparagine (ASN) residues during the 100 ns simulation. There were also two negative (glutamic acid, GLU) and two positively charged interactions (arginine, ARG) with a greater number of interactions with GLU for 92%, 93%, and 99% of the time. When the pressure was increased there was a significant decrease in interactions with only one polar interaction with serine (SER) 52% of the simulation time, one hydrophobic interaction with phenylalanine (PHE) 42% of the time, and one interaction with a positively charged lysine (LYS) 55% of the time (Supplementary Fig. S1a,b).

Ampicillin interacted with positively charged arginine (ARG) 97%, and 73% of the time while most other interactions were with asparagine (ASN) and glutamine (GLN) both polar interactions, and aspartic acid (ASP) a negatively charged interaction, and for only 30% of the simulation time. Ampicillin under increased pressure interacted with ARG 100% of the time and only had one interaction under 60% of the time with SER. There was one negatively charged interaction with ASP for 73% of the simulation time and one hydrophobic interaction with alanine (ALA) 69% of the simulation time. There were also polar interactions with ASN, and GLN for more than 95% of the simulation time (Supplementary Fig. S1c,d).

Sulfamethoxazole-trimethoprim interacted with a higher amount of ionic bonds, but the ligand visibly moved to a different binding location during the simulation. No interactions were above 51% of the simulation time but when the pressure was increased, the interaction fraction increased by > 50% to the same residues. And a new interaction between ARG for more than 107% of the time same type, showing multiple contacts. The increased binding could have caused the SXT to bind tightly to the new region of the AcrB protein and not expelled by the efflux pump (Supplementary Fig. S1e,f).

### Structural changes

**Figure 3:**
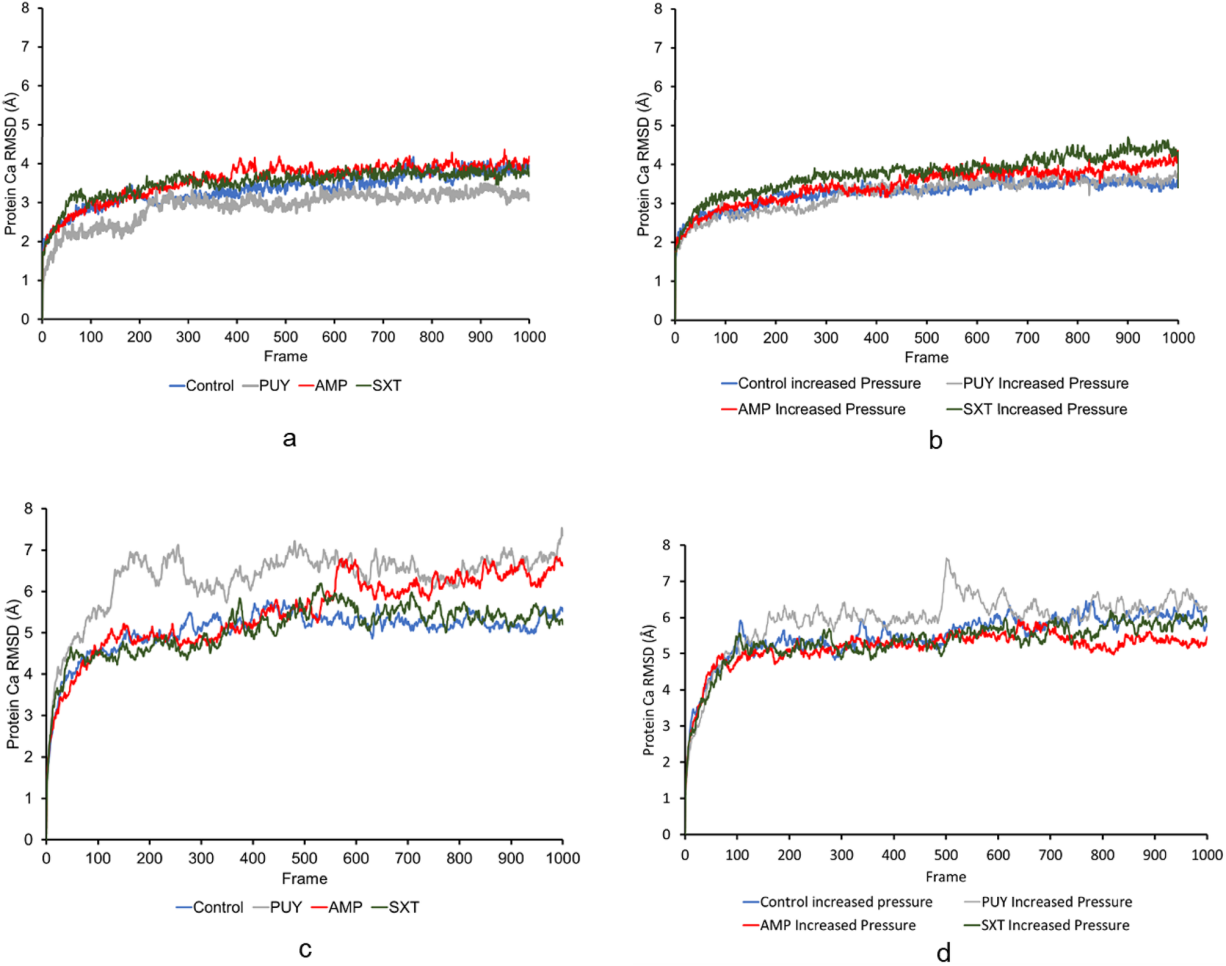
Shows the RMSD values of 100 ns for (**a**) standard pressure and (**b**) increased pressure and 10 ns simulations for (**c**) standard pressure and (**d**) increased pressure. RMSD values higher on average during all standard pressure simulations compared to increased pressure simulations indicate more structural changes in proteins simulated under standard pressure conditions. This is likely because the proteins operating under pressure cannot move as frequently to maintain homeostasis of the cell.

The average RMSD values for the 100 ns simulations were 1.646 ± 0.958 Å, 1.640 ± 0.801 Å, 1.788 ± 1.01 Å, 1.713 ± 0.972 Å for Control, PUY, AMP, and SXT respectively shown in Fig. 3a and c. The average RMSD value for the 100 ns simulations are shown in Fig. 3b and d with increased pressure were 1.320 ± 0.631 Å, 1.384 ± 0.595 Å, 1.436 ± 0.650 Å, 1.370 ± 0.608 Å for Control, PUY, AMP, and SXT respectively. The simulations on average were stable around 70 ns.

**Figure 4:**
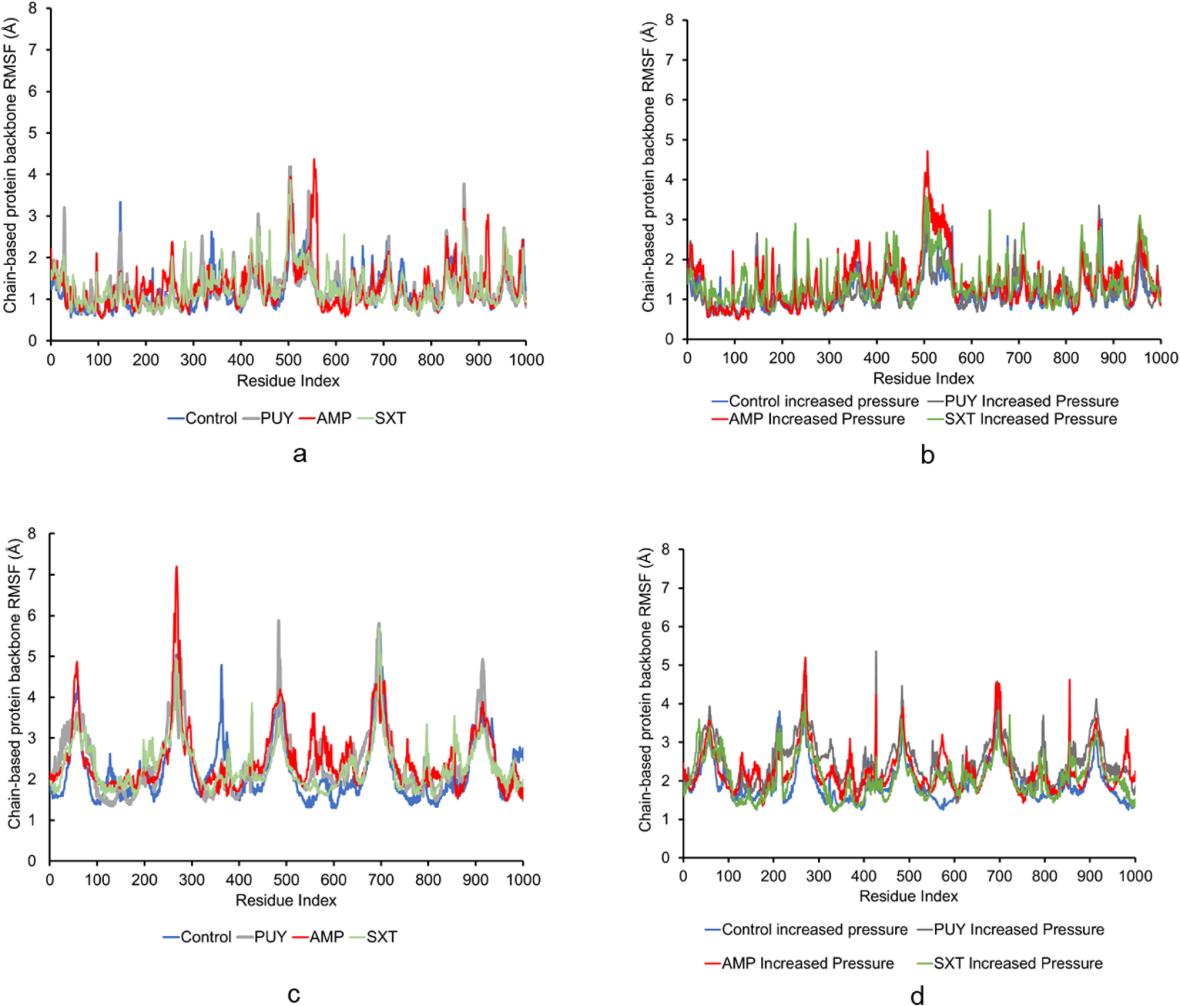
Shows the RMSF values of 100 ns for (**a**) standard pressure and (**b**) increased pressure and 10 ns simulations for (**c**) standard pressure and (**d**) increased pressure. PUY and AMP had the highest flexibility under standard conditions and increased pressure simulations with the greatest flexibility in standard pressure simulations of the full AcrAB-TolC complex. While the highest values were in standard pressure simulations, each simulation had the most flexibility in one common residue, MET 1.

The Cα RMSF which measure the flexibility and stability of protein residues were 1.320 ± 0.631, 1.384 ± 0.595, 1.436 ± 0.650, 1.370 ± 0.608 for Control, PUY, AMP, and SXT respectively, shown in Fig. 4a and c. The Cα RMSF which measure the flexibility and stability of protein residues with increased pressure were 1.233 ± 0.486, 1.403 ± 0.663, 1.325 ± 0.575, 1.387 ± 0.681 for Control, PUY, AMP, and SXT, respectively and are shown in Fig. 4b and d. The simulations, on average, stabilized around 8 ns. The residues that consistently had high flexibility in standard and increased pressure simulations were methionine (MET) 1 and GLU 377, likely because they were a part of isolated segments of the protein that had independent edges of other chains and fewer interactions with other amino acids.

**Figure 5:**
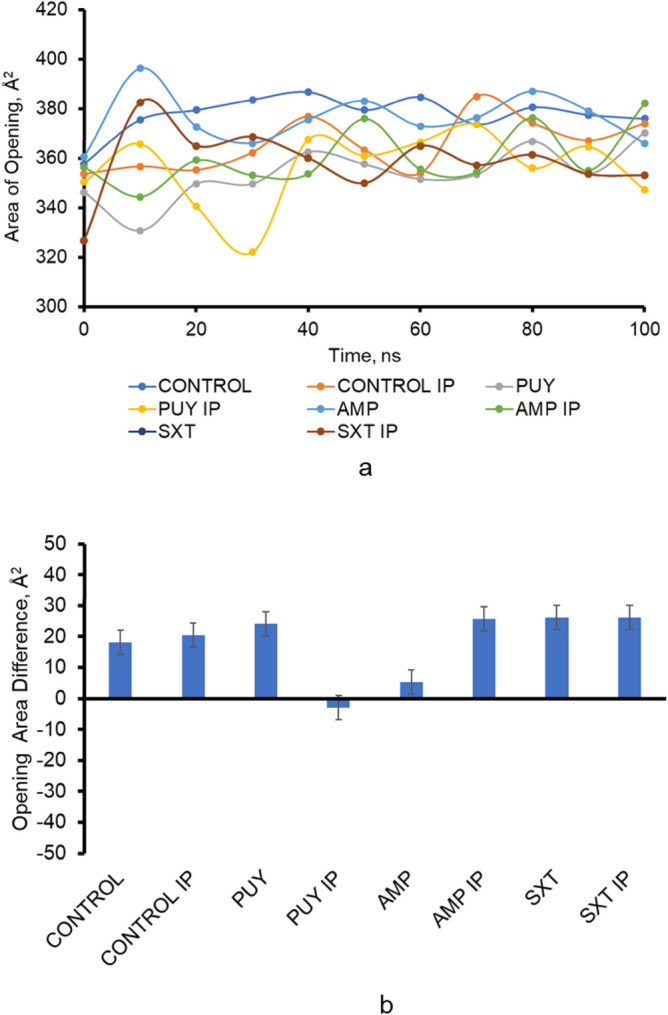
Shows (**a**) the area of the opening on top of the AcrB protein leading to the AcrA protein over the duration of the 100 ns simulation and the (**b**) difference in the area of the opening from the beginning to the end of the simulation. This opening compared to the opening of the TolC protein was more stable showing that larger changes occur in the TolC protein than in the AcrB protein when comparing simulations with and without a ligand present.

The area of the opening of the top of the AcrB protein was measured over the course of each simulation between residues D-47 on Chains A, B, and C. Figure 5 shows the changes over the 100 ns simulation. While the MM-GBSA scores to calculate the free energy of ligand–protein binding for SXT are higher, there is a smaller area of opening than for AMP and PUY tests. The distance opened at the top of the AcrB was significant in the following cases.

There was a significant difference in the mean area of opening between control simulations and the control simulated under increased pressure (*p* value = 0.003), similar to AMP and AMP under increased pressure (*p* value = 0.002). There was also a significant difference in the mean area between control and PUY, AMP, and SXT simulations, while under increased pressure, there was only a significant difference between the control and PUY tests (*p* value < 0.05).

On average, the change in the area of the opening was 18.443 Å^2^ and 17.340 Å^2^ for standard simulations and increased pressure simulations, respectively. The average area was 366.495 for standard simulations and 360.153 for simulations under increased pressure.

The area between the six aspartate residues D-371 and D-374 was measured in each 10 ns simulation of the AcrAB-TolC complex. The differences in the area were taken after the protein stabilized due to the simulation starting in an open state compared to the remainder of the simulation efflux pump. Figure 5 shows the final distance of the AcrB opening after full efflux pump simulations. Figure 5 shows the change in the area of the AcrB opening from before and after stabilization.

Interestingly, the opening under pressure was usually smaller than the standard pressure simulations. But there was movement in the opening under pressure. On average, the change in the area of the opening was − 2.797 Å^2^ and 4.932 Å^2^ for standard simulations and increased pressure simulations, respectively. The average area was 153.975 for standard simulations and 173.838 for increased pressure simulations.

**Figure 6:**
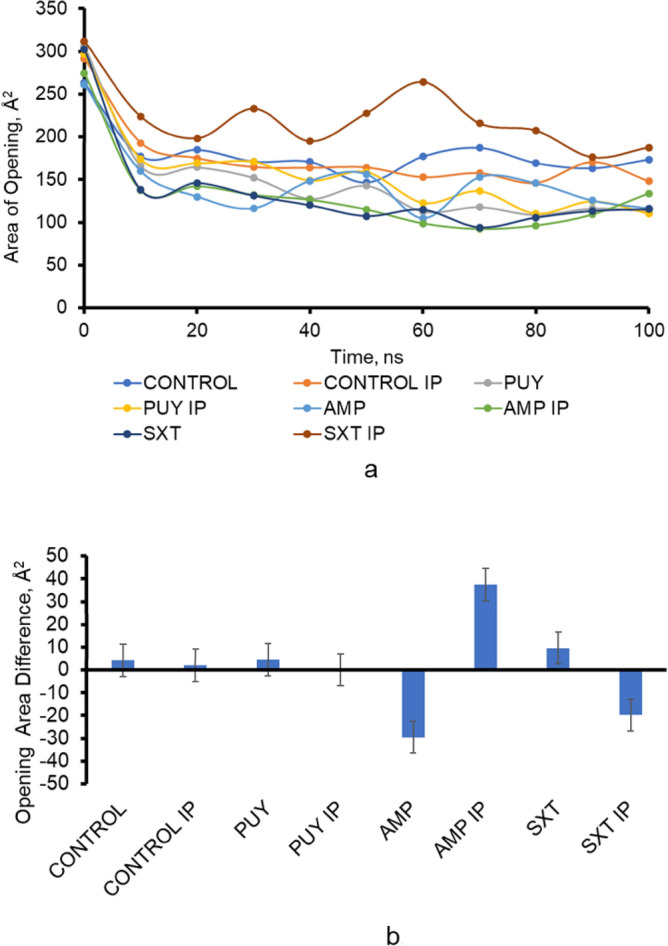
Shows (**a**) the area of the opening of TolC over the duration of the 10 ns simulation and (**b**) the difference in the area of the opening from the beginning to the end of the simulation. The largest change in opening occurred with AMP simulated under increased pressure while SXT simulated under increased pressure had the largest opening throughout the simulation. The increase of resistance was validated by experimental data^21^.

**Figure 7:**
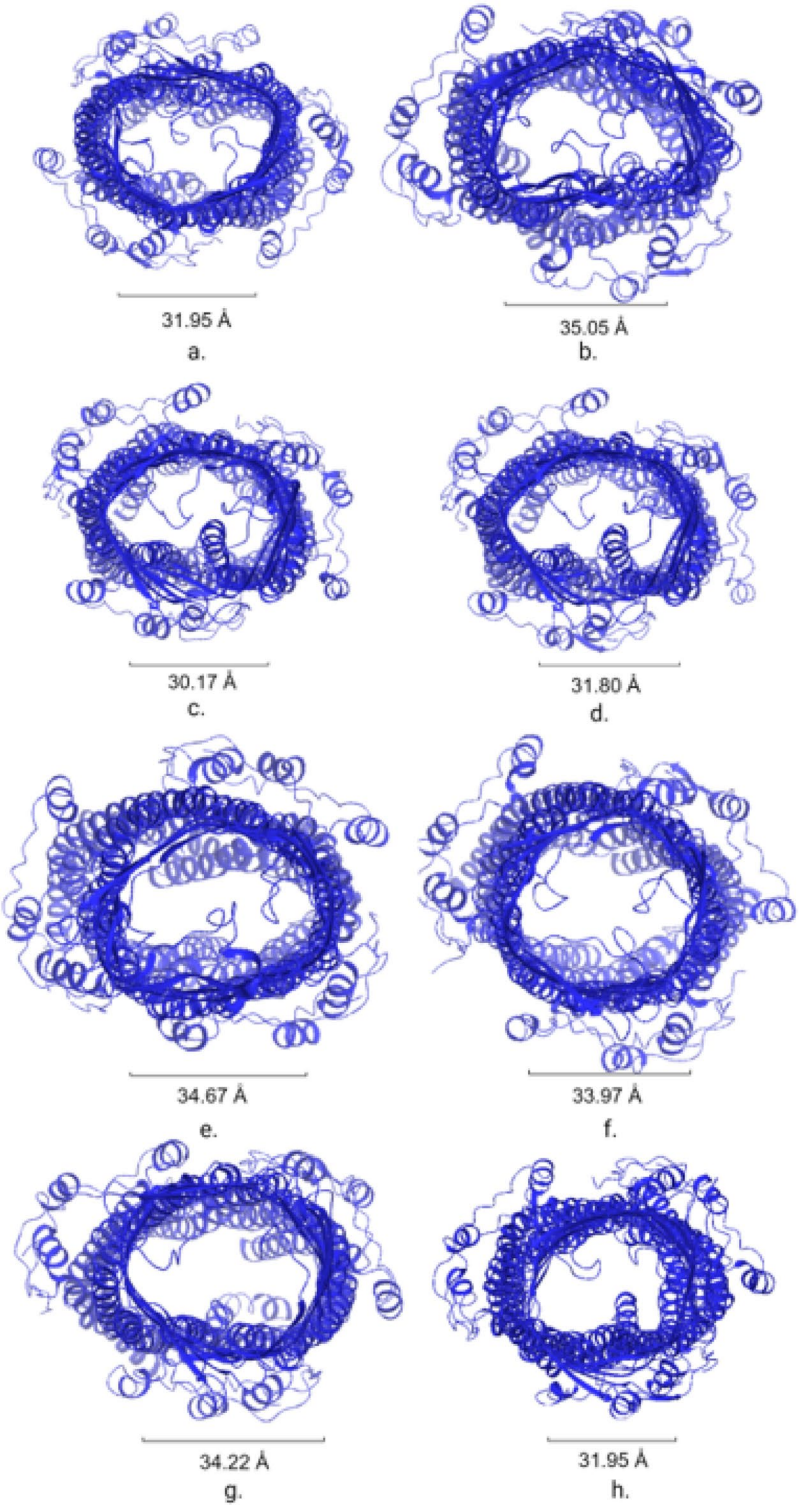
Shows the ending position of the top of the TolC protein of the efflux pump of simulations (**a**) Control, (**b**) Control increased pressure, (**c**) PUY, (**d**) PUY increased pressure, (**e**) AMP, (**f**) AMP increased pressure, (**g**) SXT, (**h**) SXT increased pressure. The largest area of openings throughout the simulations belonged to proteins in SXT simulations. The largest openings are followed by Control increased pressure, AMP increased pressure showing that the opening of increased pressure tests were larger than standard pressure tests for all cases except for PUY.

A ligand in which experiments showed the most resistance (AMP) had the largest change of 37.459 Å^2^. The only significant changes in distance for the TolC opening were between AMP and AMP under increased pressure and SXT and SXT under increased pressure, shown in Fig. 6, with a *p* value of 4.61e−8 and 8.53e−5, respectively (both *p* value < 0.05). The opening size was significant between the ligand and the control under increased pressure conditions for AMP (*p* value = 1.0e−4) and SXT (*p* value = 5.0e−4). These statistical results showed the distance the TolC opened was not a significant factor in resistance, especially compared to the ligand interactions, stability, and binding free energy. Visualization of the TolC opening at the end of each simulation is shown in Fig. 7. The largest change in opening of TolC, occurred in AMP simulations under increased pressure shown also in Fig. 7f.

Notably, none of the simulated ligands caused resistance to control bacteria samples that were not treated with stress. While there was a larger opening and strong binding, the bacteria did not express resistance until after they were exposed to environmental stressors. This is most likely because the bacteria enacted resistance mechanisms to alleviate the stress of aerosolization and continued to utilize resistance mechanisms after stress. Many factors are involved in resistance and efflux pump resistance. These studies can be built upon to delineate the pathways of the ligands after aerosolization.

### Prime MM-GBSA analysis

**Figure 8:**
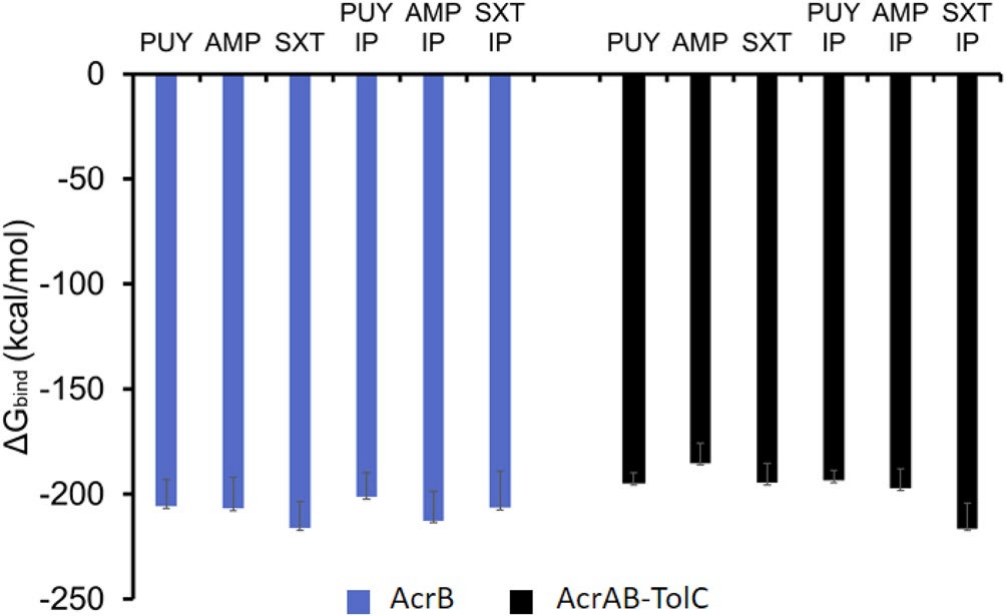
Shows the ΔG_bind_ MM-GBSA scores in kcal/mol of the 12 simulations run with ligands, PUY, AMP and SXT. 100 ns AcrB simulations are represented by blue bars and AcrAB-TolC 10 ns simulations are represented by black bars. The only significant difference found was between SXT simulations in the AcrB and AcrAB-TolC proteins under standard pressure. This showed there was no direct correlation between the MM-GBSA score of an individual efflux pump and the experimental antibiotic resistance expressed. But there was increased efflux pump activity in *E. coli* cells which could have affected the antibiotic resistance rather than the routine functioning of the individual efflux pumps.

The average differences across antibiotics were not significant under each condition when comparing PUY, AMP, and SXT. However, there was a significant difference between SXT under normal vs increased pressure, with a *p* value of 0.03. While the MM-GBSA score was lower in PUY under increased pressure conditions, there was overexpression of AcrAB-TolC efflux pump activity and a heightened stress response in experimental data. This shows that the MM-GBSA score and potency of the ligand against the efflux pump are not working solely as the antibiotic resistance response. These behaviors are working with other mechanisms to develop antibiotic resistance. The MM-GBSA scores for each simulation are shown in Fig. 8. When comparing subsets of simulations, SXT had the strongest free energy based on the MM-GBSA score. This is most likely due to the SXT ligand moving to a different binding pocket during simulations. The SXT ligand moved binding sites and was bound more tightly to its area of interest, approximately 15 Å away from the binding pocket. While SXT had stronger MM-GBSA scores *E. coli* cells were susceptible, showing that MM-GBSA scores without other analyses such as RMSD, RMSF, and structural changes in the protein do not give a comprehensive view into what occurs when *E. coli* cells are stressed and introduced to antibiotics.

## Discussion

This research was conducted to build upon research conducted by Smith and King^11,21^. The objective was to visualize the interactions of the AcrAB-TolC efflux pump and antibiotics on a molecular level under standard and increased pressure conditions. Researchers found a change in gene expression of the *marR* gene encoding the MarR protein repressor. Smith and King^11^ showed that the fold gene expression of the *marR* gene changed up to ~ 14 and as low as ~ 5 times as a result of aerosolization. These bacteria were aerosolized for five different time durations. The results showed that the level of expression of tested antibiotic resistance genes (ARGs) changed when exposed to different durations of aerosolization stress. While a laboratory strain (*E. coli* MG1655) was used in this case to track changes, clinical strains have shown MarR mutations in the real world^37^. This research was conducted to gather baseline data to show aerosolization's effect on bacterial cell behavior, including phenotypic and genotypic changes. These changes indicate there was overactivity of efflux pumps to withstand the stressors imposed on *E. coli* cells by aerosolization stress. To further analyze the effects of aerosolization stress on *E. coli* cells, Smith and King^21^ collected airborne *E. coli* using a high-volume, low cutpoint wetted wall cyclone bioaerosol collector with a pressure drop of 55″ H_2_O. The bacteria were also tested for antibiotic susceptibility, and results showed that aerosolized *E. coli* cells that experience the 55″ H_2_O pressure drop started out susceptible to seven of the nine antibiotics tested (AMP, CF, GM, IPM, CIP, PUY, SXT) with intermediate resistance to TE, and resistance to CFP. After aerosolization, *E. coli* cells showed unanimous resistance to AMP, CF, and PUY, maintaining susceptibility to GM, IPM, CIP, and SXT, intermediate resistance to TE, and resistance to CFP^11,21^.

The docking scores and resistance after aerosolization were only comparable for IPM (susceptible) and PUY (resistance) under the assumption that a better binding affinity with a more negative value would result in efflux of the antibiotic and resistance. *E. coli* cells were susceptible to IPM which had the highest Emodel score and *E. coli* cells were resistant to PUY which had the lowest Emodel score. Some cases such as CIP that had a high docking score but *E. coli* cells were susceptible, this is an indication that resistance is not solely correlated to docking scores. There are many other factors within the *E. coli* cells contributing to resistance, and this information shows that while CIP may have been able to bind to the efflux pump’s binding site, there were other reasons why the antibiotic was still able to kill the cells. Interestingly the most resistance was found against cell wall synthesis inhibitors (AM and CF). Supplementary materials show a more detailed visualization of ligand docking to the AcrB binding site (Supplementary Fig. S1). Interestingly PUY and AMP were the only two ligands simulated with polar interactions. These interactions show a relationship with the pump opening. In the case of PUY, there were more polar interactions in the standard pressure simulation which had a greater area of opening as compared to the increased pressure simulations. The opposite was true for AMP simulations, in which there were more polar interactions in nearly 100% of the simulation time in increased pressure simulations, which displayed a larger opening of the efflux pumps. This pattern was not seen in SXT simulations as the ligand moved to a different binding pocket. While binding increased by ~ 50%, the pump closed, indicating the strong binding did not allow for a release of the ligand.

AMP, CF, CFP and IPM are cell wall synthesis inhibitors, PUY, GM and TE are protein synthesis inhibitors and CIP, and SXT are DNA synthesis inhibitors^38–40^. Smith and King^11^ observed more resistance responses to cell wall synthesis inhibitors targeting one of the cell’s first lines of defense. It was expected to see less flexibility in simulations conducted under increased pressure because of the inability to move under heightened pressure on the protein and on a larger scale on the *E. coli* cells being aerosolized. There was more flexibility in simulations conducted under standard pressure for the AcrB protein and the full tripartite complex AcrAB-TolC, showing that increased pressure causes more rigidity in the efflux pump most likely in the real world to maintain homeostasis within the cell.

During simulations, it was evident that the SXT ligand moved and bound to another binding pocket approximately 15 Å away from the binding site. This could have affected the MM-GBSA score and the distance the TolC opening was opened. MM-GBSA scores were taken for at least 20 frames per simulation and analyzed. The binding after the simulation seemed to be stronger according to the stronger ΔG_bind_. There was not a significant difference between the MM-GBSA scores, but there was a difference in the opening and closing of the efflux pump after binding. Compared to the control and PUY samples, the AMP increased pressure simulation resulted in the largest opening, while the SXT increased pressure simulation resulted in the smallest.

While we cannot conclude from this study that resistance is directly linked to binding affinity, binding energy, and protein flexibility, we can visualize some relationships between docking location, binding affinity, and resistance. The ability to model drugs as screening methods has proven helpful in drug discovery. This study has also shown the complicated nature that allows bacterial cells to become resistant to drugs in simulating one of the many ways cells evade the effect of antibiotics. This information can be used in the future when designing EPIs to avoid antibiotics that bind well to the efflux pumps.

The collection of the data has shown that while there were some correlations, MD simulation results alone cannot predict the resistance expression of bacteria. While they can be used to inform the interaction of drugs with the efflux pump to further develop efflux pump inhibitors (EPI) or improve upon existing drugs, they cannot determine the other acquired, adaptive, or intrinsic resistance antibiotic resistance mechanisms, bacterial cells can activate to evade drugs. Considering many known and unknown methods of *E. coli* cells to evade the effects of antibiotics, this study adds more insight into how efflux pump affects antibiotic resistance.

## Conclusion

This study has shown some of the changes occurring on a molecular level when airborne bacteria are exposed to stress and antibiotics to build upon previous experimental research^11^. This is not a comprehensive analysis of the changes in *E. coli* after being aerosolized or exposed to antibiotics. There is a need to monitor real-time antibiotic resistance responses as evolving bacteria and their resistance responses cannot be overstated and have not been uncovered comprehensively^41^. Through this study, we gained valuable insights into the multifaceted nature of bacterial resistance. The study's exploration of factors such as RMSD and RMSF and the significant MM-GBSA scores associated with specific ligands like SXT signify the complexity of antibiotic resistance mechanisms. There was more flexibility in simulations conducted under standard pressure for the AcrB protein and the full tripartite complex AcrAB-TolC, showing that increased pressure causes more rigidity in the efflux pump mostly likely in the real world to maintain homeostasis within the cell. Resistance in experimental data shows there is some relationship between the docking scores and ligand–protein interactions. MM-GBSA, structural changes must be taken into account with other cellular responses such as other OM proteins, and mutations of targets within the cell, to delineate antibiotic resistance mechanisms. These discoveries emphasize the urgency of further research and surveillance efforts in understanding and combating antibiotic resistance in real-time scenarios, which is crucial in our ongoing battle against this global health threat. The antibiotic and ligand that *E. coli* cells were most resistant to was AMP, and this ligand had one of the best docking scores, the most area for opening the AcrB protein, and the strongest percentage of interactions throughout the 100 ns simulations. The average RMSD values were higher for proteins in cases. The MM-GBSA scores were the strongest for SXT, the only ligand that moved binding pockets. While the MM-GBSA scores were stronger, the opening of the protein was smaller, and the binding site was not initialized.

## Supplementary Information

Supplementary Information.

## Data Availability

The datasets used and/or analyzed during the current study are available from the corresponding author on reasonable request.
