# Publisher Correction: *Escherichia coli* resistance mechanism AcrAB-TolC efflux pump interactions with commonly used antibiotics: amolecular dynamics study

**DOI:** 10.1038/s41598-024-80622-9

**Published:** 2024-12-02

**Authors:** Brooke L. Smith, Sandun Fernando, Maria D. King

**Affiliations:** Aerosol Technology Laboratory, Biological and Agricultural Engineering Department, Texas A&M University, College Station, TX 77843 USA

Correction to: *Scientific Reports* 10.1038/s41598-024-52536-z, published online 01 February 2024

The Acknowledgements section in the original version of this Article was omitted. It now reads:

“This work was funded, in part, by the grant from the Hatch Program, United States Department of Agriculture, National Institute of Food and Agriculture (USDA NIFA): TEX09746, National Science Foundation (NSF): CBET 2034048, and DHHS-NIH-National Institute of Allergy and Infectious Diseases (NIAID): 5R21AI169046-02.”

The original Article has been corrected.

