# Supplementary material for: Escherichia coli resistance mechanism AcrAB-TolC efflux pump interactions with commonly used antibiotics: a molecular dynamics study
Source: Sci Rep. 2024 Feb 1;14:2742. doi: 10.1038/s41598-024-52536-z (PMC10834472; doi:10.1038/s41598-024-52536-z)
Supplement: Supplementary file 1 — Supplementary Information. [file 41598_2024_52536_MOESM1_ESM.docx]

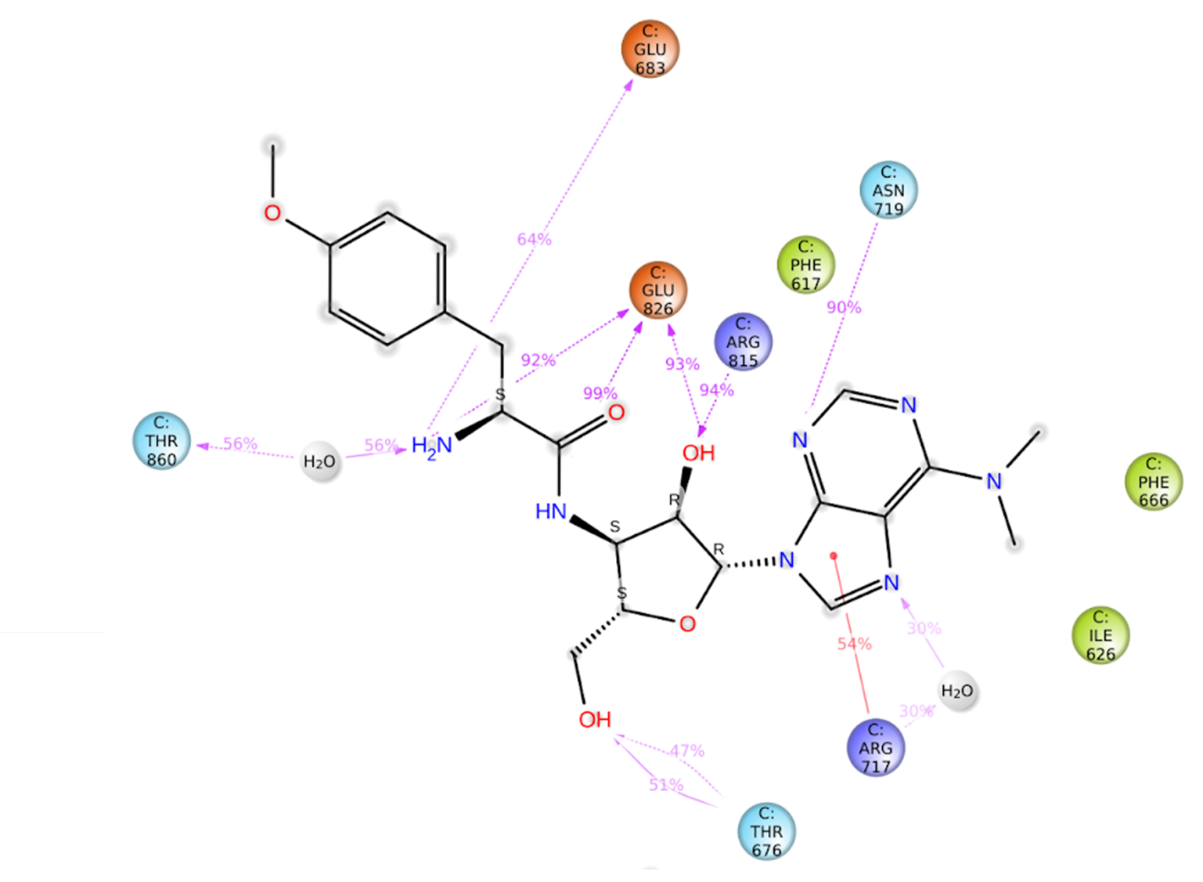


a.


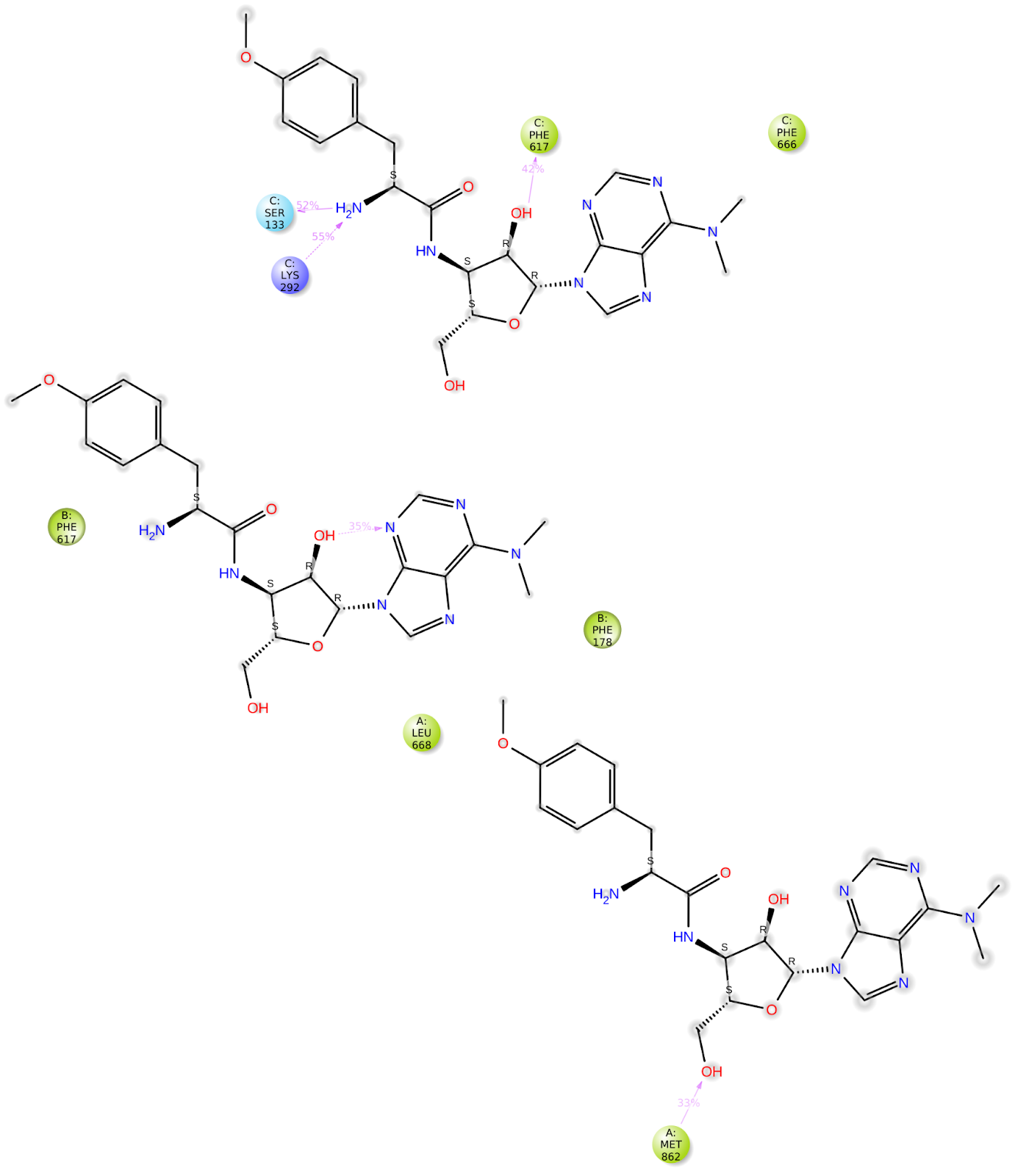


b.


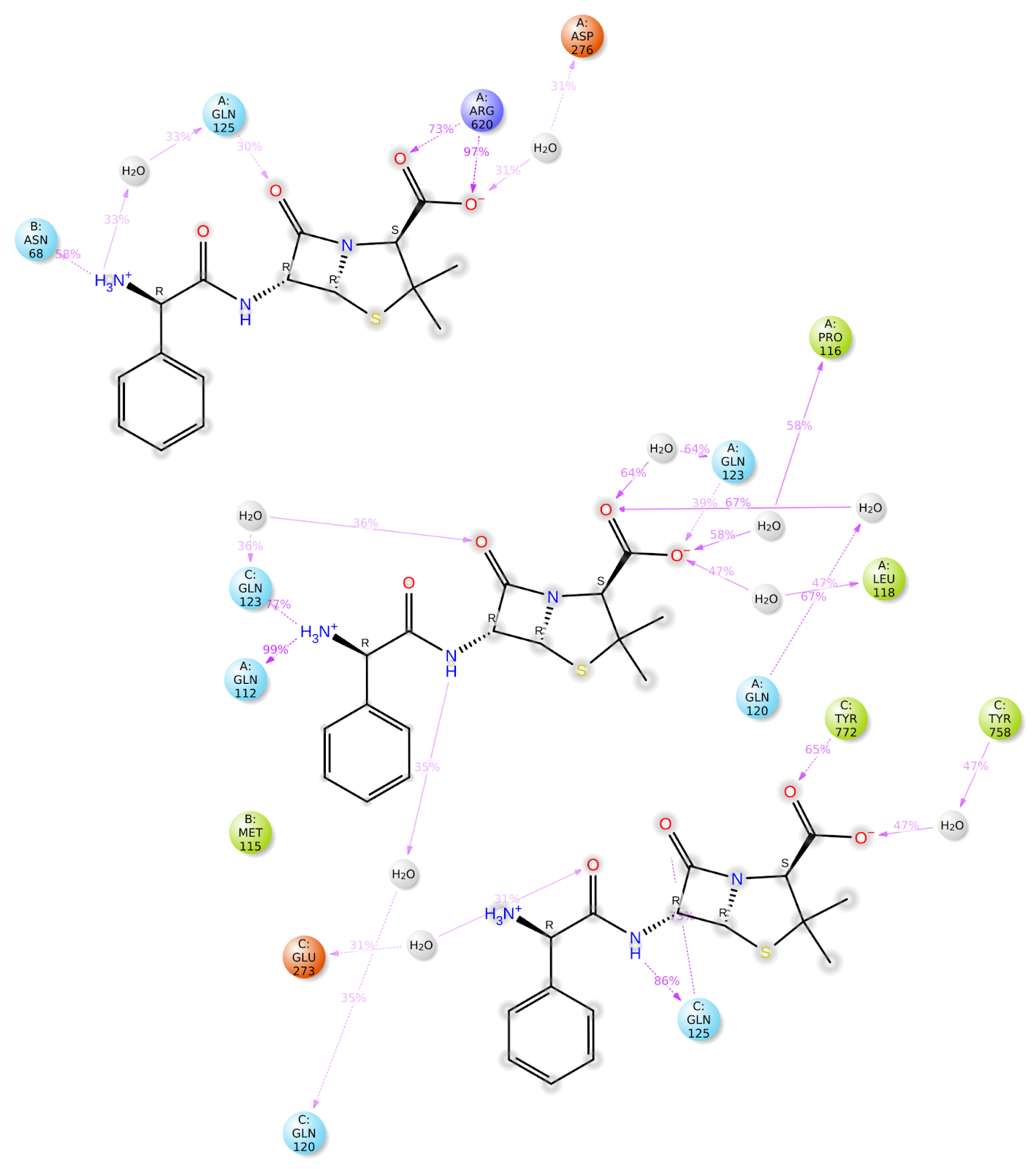


c.


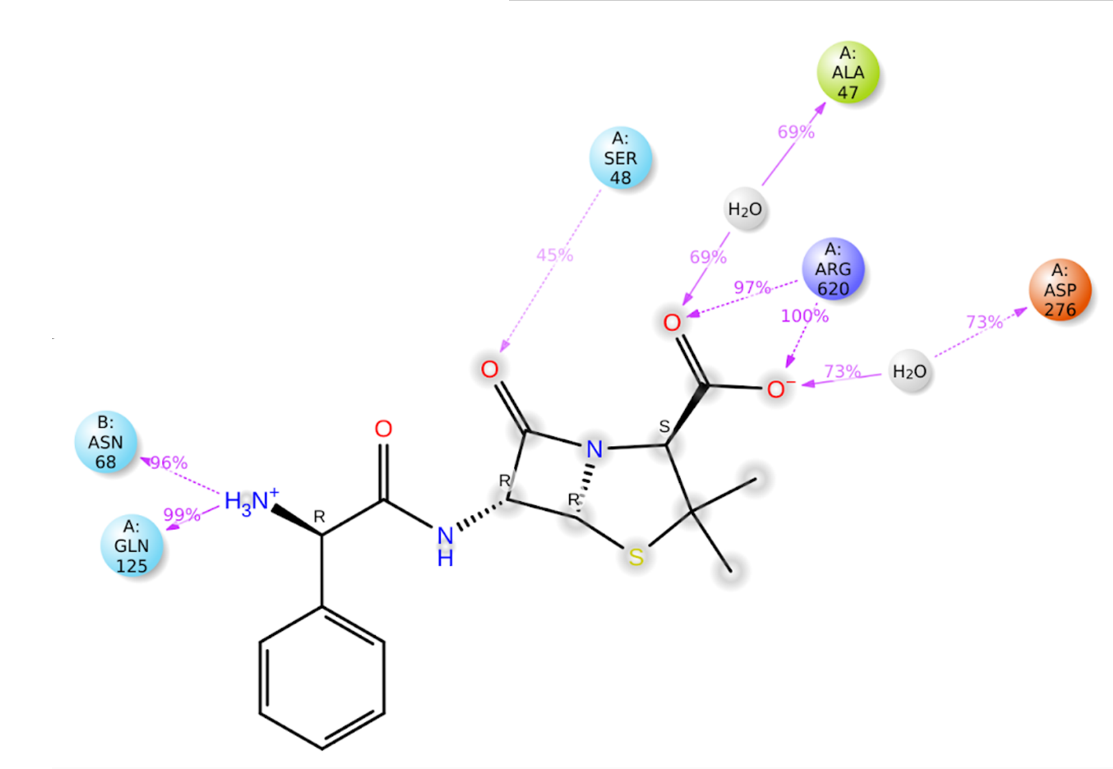


d.


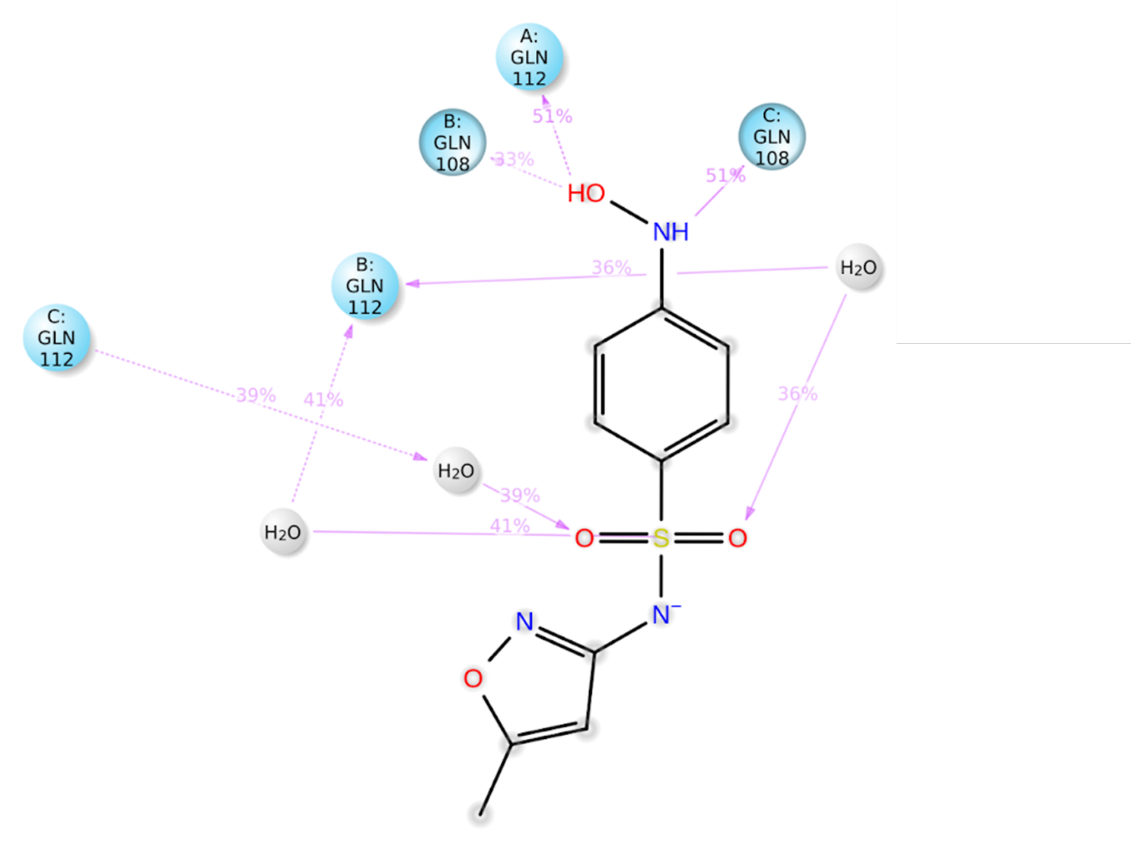


e.


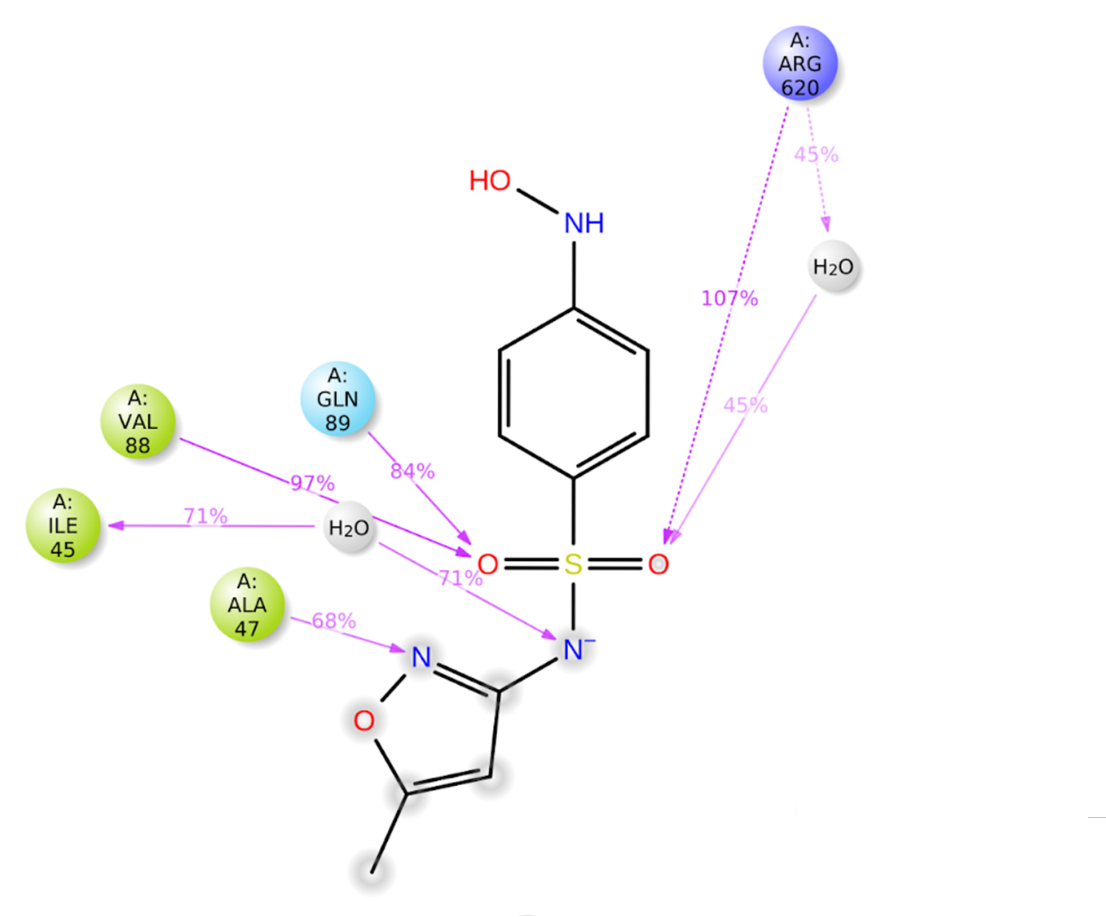


f.

**S.1.** Ligand interaction diagrams of a) PUY in standard pressure, b) PUY in increased pressure, c) AMP in standard pressure d) AMP in increased pressure, e) SXT in standard pressure f) SXT in increased pressure
